# Host-Determined Diversity and Environment-Shaped Community Assembly of Phyllosphere Microbiomes in Alpine Steppes Ecosystems

**DOI:** 10.3390/microorganisms13061432

**Published:** 2025-06-19

**Authors:** Kaifu Zheng, Xin Jin, Jingjing Li, Guangxin Lu

**Affiliations:** College of Agriculture and Animal Husbandry, Qinghai University, Xining 810016, China; zhengkf@qhu.edu.cn (K.Z.); 18894310895@163.com (X.J.); lijingjing1879705@163.com (J.L.)

**Keywords:** alpine steppes, phyllosphere microbiomes, diversity, community assembly

## Abstract

The Qinghai–Tibet Plateau is a key region for biodiversity conservation, where alpine grasslands are ecologically important. While previous studies have mainly addressed vegetation, ecosystem processes, and soil microbes, phyllosphere microorganisms are essential for nutrient cycling, plant health, and stress tolerance. However, their communities remain poorly understood compared to those in soil. The relative influence of host identity and environmental conditions on shaping phyllosphere microbial diversity and community assembly remains uncertain. In this study, we characterized phyllosphere bacterial and fungal communities of the phyllosphere at two alpine steppe sites with similar vegetation but climatic conditions: the Qilian Mountains (QLM) and the Qinghai Lake region (LQS). At both sites, *Cyanobacteriota* and *Ascomycota* were the predominant bacterial and fungal taxa, respectively. Microbial α-diversity did not differ significantly between the two regions, implying that host-associated mechanisms may stabilize within-site diversity. In contrast, β-diversity exhibited clear spatial differentiation. In QLM, bacterial β-diversity was significantly correlated with mean annual precipitation, while fungal α- and β-diversity were associated with soil nutrient levels (including nitrate, ammonium, available potassium, and phosphorus) and vegetation coverage. At LQS, the β-diversity of both bacterial and fungal communities was strongly influenced by soil electrical conductivity, and fungal communities were further shaped by vegetation cover. Community assembly processes were predominantly stochastic at both sites, although deterministic patterns were more pronounced in QLM. Variability in moisture availability contributed to random bacterial assembly at LQS, while increased environmental heterogeneity promoted deterministic assembly in fungal communities. The elevated diversity of microbes and plants in QLM also reinforced deterministic processes. Overall, our findings support a host–environment interaction hypothesis, indicating that host factors primarily govern α-diversity, while climatic and soil-related variables have stronger effects on β-diversity and microbial assembly dynamics. These insights advance our understanding of how phyllosphere microbial communities may respond to environmental change in alpine ecosystems.

## 1. Introduction

The Qinghai–Tibet Plateau is a globally important region for biodiversity conservation, with alpine steppes occupying a central ecological role in sustaining ecological balance [1,2]. These ecosystems are vital for maintaining regional water retention, carbon sequestration, and livestock productivity [3,4]. Previous studies have primarily focused on the spatial distribution patterns [5], functional roles [6], and degradation dynamics [7] of alpine steppe vegetation, along with investigations of soil microbial communities [8] and greenhouse gas emissions [9]. In recent years, research has increasingly shifted toward another ecologically significant but historically underexplored component: phyllosphere microorganisms. These microbial assemblages, which inhabit leaf surfaces, exhibit remarkable taxonomic diversity and fulfill critical ecological functions, including nitrogen fixation, enhancement of plant growth, and inhibition of pathogenic organisms [10]. Consequently, they have emerged as a growing focus in studies of alpine microbial ecology.

The total leaf area of terrestrial plants has been estimated to reach approximately 1 × 10^8^ km^2^, positioning it among the largest biological interfaces on the planet [11]. On average, each square centimeter of leaf surface hosts between 10^6^ and 10^7^ bacterial cells, contributing to a global phyllosphere microbial population approaching 10^26^ cells [12]. These microorganisms inhabiting leaf surfaces perform crucial ecological functions, such as fixing atmospheric nitrogen and solubilizing phosphorus, thereby supporting plant growth [13,14]. Recent research has increasingly acknowledged that the structure of phyllosphere microbial communities is largely shaped by host plant filtering processes [15]. For example, the phyllosphere of rice is commonly dominated by members of *Bacteroidota* and the genus *Bacillus* [16], while *Pseudomonas* from the phylum *Proteobacteria* is frequently enriched in the maize phyllosphere [17]. Environmental conditions also play a critical role in influencing shifts in microbial community composition [18]. Under drought stress, for instance, the dominant bacterial taxa in the wheat phyllosphere tend to shift from *Proteobacteria* to *Firmicutes* [19]. Furthermore, microbial communities associated with different Tamarix species are generally more similar within the same environment, while individuals of a single species growing in distinct climates often harbor compositionally different communities [20]. These observations underscore the importance of clarifying the relative influence of host plant identity and environmental variables in shaping phyllosphere microbial community assembly, which remains a central question in plant–microbe interaction research.

The Qinghai–Tibet Plateau is noted for its extreme elevation, intense ultraviolet radiation, and severe cold, conditions that together impose heavy ecological stress on local ecosystems [21,22,23]. From 2000 to 2020, substantial interannual fluctuations in precipitation and temperature were recorded across the region, and these climatic shifts strongly altered indices of ecological degradation [24]. Studies of alpine soil microbiomes further indicate that community assembly depends on both vegetation identity and abiotic factors, although the influence of each driver changes with spatial scale and environmental setting [25,26,27]. In the phyllosphere of alpine steppes, a central unanswered question is whether community assembly is governed primarily by host identity or by abiotic factors along the Plateau’s sharp environmental gradients. Progress is constrained by two issues. First, alpine steppe vegetation is highly diverse and patchily distributed, which complicates the regional standardization of host backgrounds [28]. Second, large variations in climate, topography, soil properties, and local land use create pronounced environmental heterogeneity [29]. As a result, phyllosphere surveys in these systems remain limited, and the relative contributions of host and environment to microbial diversity and assembly at the plant-community scale are still unquantified.

To address this issue, we chose two alpine steppe localities, the Qilian Mountains (QLM) and the Qinghai Lake margin site (LQS), on the basis of ten-year climatic records. Although the sites differ substantially in mean annual precipitation and temperature, their vegetation is largely comparable. We posited that pronounced differences in microbial diversity between the sites would indicate that environmental filtering predominates, whereas similarity would imply a stronger role for host identity. To evaluate this proposition, we characterized community-level phyllosphere microbiota by high-throughput sequencing and asked three questions: (i) What taxa compose the phyllosphere microbiome at each site? (ii) How do bacterial and fungal α and β diversity vary, and which environmental or host attributes correlate with that variation? (iii) Do the underlying assembly mechanisms differ between regions, and, if so, which factors are decisive? By partitioning the relative influence of host filtering and environmental constraints, our study lays a conceptual basis for forecasting phyllosphere microbial responses to future environmental change.

## 2. Materials and Methods

### 2.1. Study Area Characteristics

**Figure 1:**
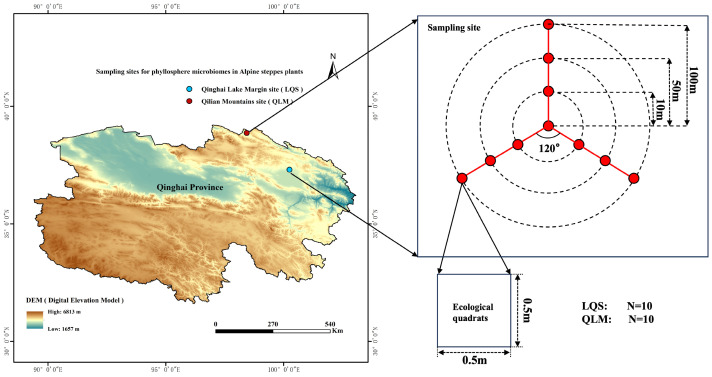
Experimental site distribution map. Note: Basemap data in the first panel sourced from the Loess plateau science data center, National Earth System Science Data Sharing Infrastructure, National Science & Technology Infrastructure of China (http://loess.geodata.cn, accessed on 5 January 2024).

This study selected two alpine steppe sites along the northern margin of the Qinghai–Tibet Plateau as representative sites for comparison (Figure 1). Both localities support comparable dominant vegetation, comprising *Stipa purpurea*, *Leymus secalinus*, *Carex tristachya*, *Oxytropis ochrocephala*, *Aster hispidus*, and *Agropyron cristatum*. Climatic conditions over the past two decades (2013 to 2023) have diverged sharply between the sites. (Table 1). The Qilian Mountains site (QLM) is located in Yanglong Township, Qilian County, Haibei Tibetan Autonomous Prefecture, Qinghai Province (98°17′45.62″ to 98°25′57.92″ E, 38°50′20.40″ to 38°55′18.68″ N), at an elevation of 3296.8 to 3498.6 m above sea level. Based on ArcGIS analyses of climate layers retrieved from the National Earth System Science Data Sharing Service Platform “http://loess.geodata.cn (accessed on 5 January 2024)” for 2013–2023, the mean annual temperature ranged from −3.64 to −2.37 °C, and annual precipitation ranged from 253.5 to 317.6 mm at this site. The Qinghai Lake margin site (LQS) is situated on the northern shore of Qinghai Lake in Gangcha County, Haibei Tibetan Autonomous Prefecture (100°14′51.60″ to 100°22′20.41″ E, 37°17′11.55″ to 37°54′10.28″ N), at an elevation of 3216.1 to 3270.8 m. Using the same dataset from the National Earth System Science Data Sharing Service Platform “http://loess.geodata.cn (accessed on 5 January 2024)” for the period 2013–2023, the 10-year average annual temperature at this site ranged from −4.75 to 0.88 °C. Annual precipitation varied from 449.4 to 484.8 mm (Table 1). Given their similar vegetation but distinct climatic conditions, these sites provide a natural platform for disentangling the relative influence of environmental filtering and host identity on phyllosphere microbial assembly in alpine steppes.

### 2.2. Site Layout, Vegetation Survey, Phyllosphere Sampling and Preservation, and Soil Sampling

In August 2023, two undisturbed summer pasture sites were selected before livestock rotation—one in QLM and the other near LQS. At each site, three transects were established at 120° intervals from a central point, with sampling points set at distances of 10, 50, and 100 m along each transect, yielding ten plots per site (Figure 1). At each sampling point, three 50 cm × 50 cm quadrats were randomly placed. All plants within the quadrats were identified and recorded, including species identity, species richness (ST), total vegetative cover (Cover), and average plant height (Height). Plant material was then collected for further analysis.

Phyllosphere material was collected at the community level: within each quadrat, every individual of every vascular plant species present was sampled, ensuring that the pooled leaf material represented the entire plant community. The second or third fully expanded, healthy leaf from the apex was clipped from each plant to yield a total sampled leaf area of approximately 200 cm^2^. Throughout the procedure, field personnel wore sterile gloves and used scissors sterilized with 75% ethanol. The collected leaves were immediately transferred into sterile, pre-labeled 250 mL centrifuge bottles containing 100 mL of sterile phosphate-buffered saline (PBS) with 0.1% Tween-80 (pH 7.0), corresponding to the quadrat ID. Each bottle was gently shaken on-site for 30 s to initiate microbial elution. Samples were then stored at −20 °C in a portable freezer, transported to the laboratory, and subsequently preserved at −80 °C until analysis.

Following vegetation and phyllosphere collection, bulk surface soil (0–20 cm) was sampled from each quadrat by extracting three diagonally spaced cores with a sterilized auger. The cores were amalgamated into a single composite, sealed in sterile polyethylene bags, and immediately chilled to −20 °C in a portable freezer. Samples were subsequently transported to the laboratory for physicochemical characterization.

### 2.3. Phyllosphere Microbial Sample Processing, Sequencing, and Bioinformatic Analysis

#### 2.3.1. Microbial Elution from Phyllosphere Samples

More than 5 g of fresh plant material was placed into a 250 mL sterile Erlenmeyer flask containing 100 mL of phosphate-buffered saline (PBS; 0.02 M, pH 7.0) supplemented with 100 μL of Tween 80. The mixture was shaken on a rotary shaker for 30 min, followed by 4 min of ultrasonication. The resulting suspension was filtered through a 0.22 μm membrane. This procedure was repeated twice. The membranes were stored at –80 °C until DNA extraction.

#### 2.3.2. DNA Extraction and Quality Assessment

Microbial DNA was extracted from the membrane filters using the FastDNA™ SPIN Kit for Soil (MP Biomedicals, Solon, OH, USA) according to the manufacturer’s instructions. DNA concentration and purity were assessed using a NanoDrop 2000 spectrophotometer (Thermo Fisher Scientific, Waltham, MA, USA). DNA integrity was evaluated by 1% agarose gel electrophoresis at 5 V/cm for 20 min.

#### 2.3.3. PCR Amplification of 16S rRNA and ITS Regions

To characterize the diversity and assembly patterns of phyllosphere microbial communities in alpine grasslands, rather than to quantify genera, we employed universal primer sets that are widely recommended for phyllosphere research [30,31]. This choice (i) maximizes the recovery of rare or yet-undescribed taxa and (ii) minimizes the taxonomic bias that genus- or species-specific primers can introduce in broad community surveys.

The V4 region of the bacterial 16S-rRNA gene was amplified with primers 515F (5′-GTGYCAGCMGCCGCGGTAA-3′) and 806R (5′-GGACTACHVGGGTWTCTAAT-3′) [30], whereas the fungal ITS2 region was amplified with primers 5.8F (5′-AACTTTYRRCAAYGGATCWCT-3′) and ITS4 (5′-AGCCTCCGCTTATTGATATGCTTAART-3′) [31].

Each 20 µL PCR contained 4 µL 5× FastPfu buffer, 2 µL 2.5 mM dNTPs, 0.8 µL of each primer (5 µM), 0.4 µL FastPfu DNA polymerase, 0.2 µL bovine serum albumin, and approximately 10 ng template DNA; nuclease-free water was added to volume. Amplification was performed on an ABI GeneAmp^®^ 9700 with the following program: 95 °C for 5 min; 25–30 cycles of 95 °C for 30 s, primer-specific annealing temperature for 30 s, and 72 °C for 45 s; final extension at 72 °C for 10 min, then hold at 10 °C prior to library preparation.

#### 2.3.4. PCR Product Verification, Purification, and Sequencing

PCR Product Verification, Purification, and Sequencing: Amplification products were examined via 2% agarose gel electrophoresis. Based on preliminary quantification, correctly sized and adequately concentrated PCR products were quantified using a QuantiFluor™-ST blue fluorescence system (Promega Corporation, Madison, WI, USA). Target bands were excised and purified using the AxyPrep DNA Gel Extraction Kit (Axygen Biosciences, Union City, CA, USA) following the manufacturer’s protocol. The purified amplicons were pooled for library preparation and subsequently sent to Lingen Biotechnology Co., Ltd. (Shanghai, China) on dry ice. Paired-end sequencing (PE300) was performed using the Illumina platform.

#### 2.3.5. Sequence Quality Control and OTU Clustering

Raw sequencing reads were subjected to quality control using fastp (version 0.20.0; https://github.com/OpenGene/fastp, accessed on 15 April 2025) [32]. The quality-filtered paired-end reads were then merged using FLASH (version 1.2.7; https://www.cbcb.umd.edu/software/flash, accessed on 15 April 2025) [33] with the following criteria: (1) reads were trimmed from the 3′ end using a sliding window of 50 bp if the average quality score within the window dropped below 20; (2) reads shorter than 50 bp or containing ambiguous bases (N) were discarded; (3) paired-end reads were merged into single sequences based on their overlapping regions, requiring a minimum overlap of 10 bp; (4) the maximum allowable mismatch ratio in the overlap region was set to 0.2; and (5) sequences were demultiplexed according to barcodes and primers, with zero mismatches allowed in barcodes and up to two mismatches permitted in primer regions, and sequence orientation was adjusted accordingly.

#### 2.3.6. OTU Assignment and Taxonomic Annotation

High-quality sequences were clustered into operational taxonomic units (OTUs) at a 97% similarity threshold using the UPARSE pipeline [34,35]. Taxonomic classification of representative sequences was performed using the RDP Classifier (version 2.2) [36]. The resulting OTU table was then used for downstream statistical analyses.

### 2.4. Statistical Analysis

Taxonomic composition at the phylum and genus levels was illustrated with stacked bar charts that retained only those taxa whose relative abundance exceeded 3%. Alpha diversity was assessed based on three indices: species richness, Shannon diversity, and phylogenetic diversity (PD), the latter was computed in R with the picante package (Faith, 1992) [37]. Differences among treatments in alpha diversity were evaluated with independent two-sample *t*-tests. Beta diversity was calculated from Bray–Curtis dissimilarities in the vegan package [38], and visualized with principal coordinate analysis (PCoA) and non-metric multidimensional scaling (NMDS). Community structures were assessed with PERMANOVA and ANOSIM, and P-values were adjusted by the Bonferroni method [39]. Finally, Mantel tests were performed to explore correlations among alpha-diversity indices (richness, Shannon, and PD), beta-diversity distance matrices, and measured environmental variables [40].

The phylogenetic normalized stochasticity ratio (pNST) was employed to identify the prevailing ecological processes shaping community assembly: values greater than 0.5 indicate that stochastic factors prevail, whereas values below 0.5 imply deterministic control [41]. To evaluate the effects of environmental predictors—including precipitation, temperature, vegetation attributes, and soil properties—on bacterial and fungal pNST values, we fitted ordinary least-squares regression models and retained only those variables with *p* < 0.05 in the final equations. All statistical analyses and data visualization were conducted in R (version 4.4.1), unless otherwise specified.

## 3. Results

### 3.1. Taxonomic Composition of Phyllosphere Microbial Communities

**Figure 2:**
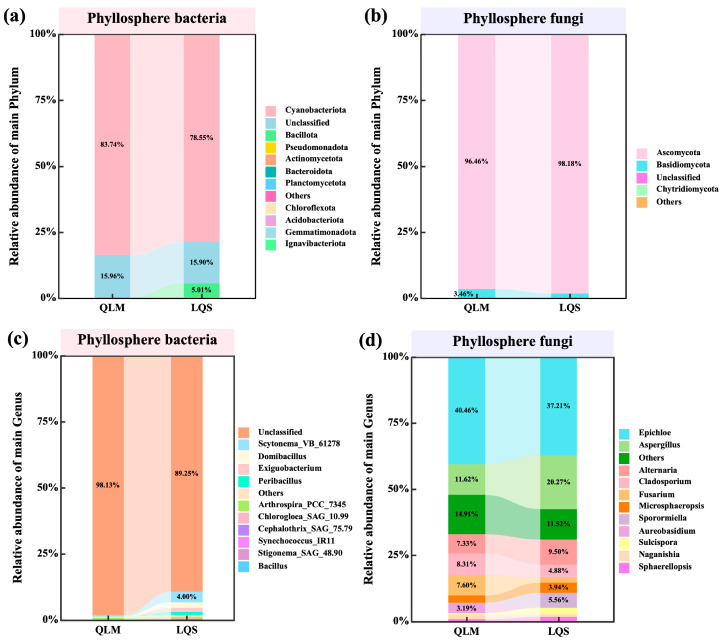
Comparative analysis of Phylum and Genus level composition in phyllosphere microbial communities between QLM and LQS. (**a**) Phyllosphere bacterial communities of main Phylum. (**b**) Phyllosphere fungi communities of main Phylum. (**c**) Phyllosphere bacterial communities of main Genus. (**d**) Phyllosphere fungi communities of main Genus. Note: Pink background in the title indicates phyllosphere bacteria; blue background indicates phyllosphere fungi.

Phyllosphere microbial communities in the alpine steppe ecosystems were profiled at the phylum and genus levels. Within the bacterial assemblage, *Cyanobacteriota* was overwhelmingly dominant, contributing 83.74% and 78.55% of the sequences at the QLM and LQS sites, respectively. Unclassified bacteria constituted a further 15.96% at QLM and 15.90% at LQS, whereas *Bacillota*, *Actinobacteriota*, and *Pseudomonadota* appeared at much lower relative abundances (Figure 2a). At the genus level, most bacterial sequences belonged to unclassified genera. Among classified taxa, dominant genera included *Scytonema*, *Domibacillus*, *Exiguobacterium*, and *Peribacillus* (Figure 2c).

Fungal communities were dominated by *Ascomycota* at both sites (QLM: 96.46%; LQS: 98.18%), with *Basidiomycota* constituting the second most abundant phylum (QLM: 3.46%; LQS: 1.75%) and Chytridiomycota barely detectable (Figure 2b). At the genus level, the predominant genera were *Epichloë*, *Aspergillus*, *Alternaria*, *Cladosporium*, and *Microsphaeropsis* (Figure 2d). Specifically, *Epichloë*, *Cladosporium*, and *Fusarium* occurred more frequently at QLM (by 3.25%, 3.44%, and 5.69%, respectively), whereas *Aspergillus*, *Alternaria*, and *Microsphaeropsis* were comparatively enriched at LQS (by 8.65%, 2.17%, and 1.21%, respectively). Although community profiles at both phylum and genus levels were broadly similar between the two grassland types, clear regional distinctions persisted, indicating that vegetation identity and local environmental conditions together modulate phyllosphere microbial communities in alpine steppes.

### 3.2. Diversity, Community Structure, and Environmental Drivers of Phyllosphere Microbiota

**Figure 3:**
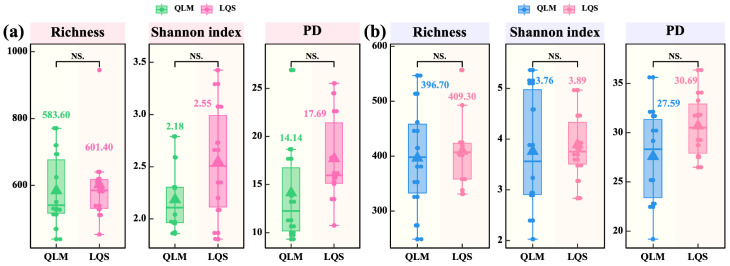
Differences in α-diversity of phyllosphere microbial communities between QLM and LQS. (**a**) Phyllosphere bacterial communities. (**b**) Phyllosphere fungi communities. The values in figures (**a**,**b**) indicate means. Pairwise comparisons among treatments QLM (*n* = 10) and LQS (*n* = 10) were conducted using independent two-sample *t*-tests (df = 18), and final P-values were adjusted using the Bonferroni correction. Note: Pink background in the title indicates phyllosphere bacteria; blue background indicates phyllosphere fungi. Asterisks indicate statistical significance (^NS.^ *p* > 0.05).

Alpha-diversity analysis showed no significant differences (*p* > 0.05) in bacterial (Figure 3a) or fungal (Figure 3b) communities between the QLM and LQS across species richness, Shannon index, and phylogenetic diversity (PD). Although LQS exhibited slightly higher and more consistent values, the differences were not statistically meaningful. These results suggest that, under similar vegetation conditions, host filtering plays a predominant role in shaping phyllosphere microbial alpha diversity, whereas environmental influence is comparatively weak.

**Figure 4:**
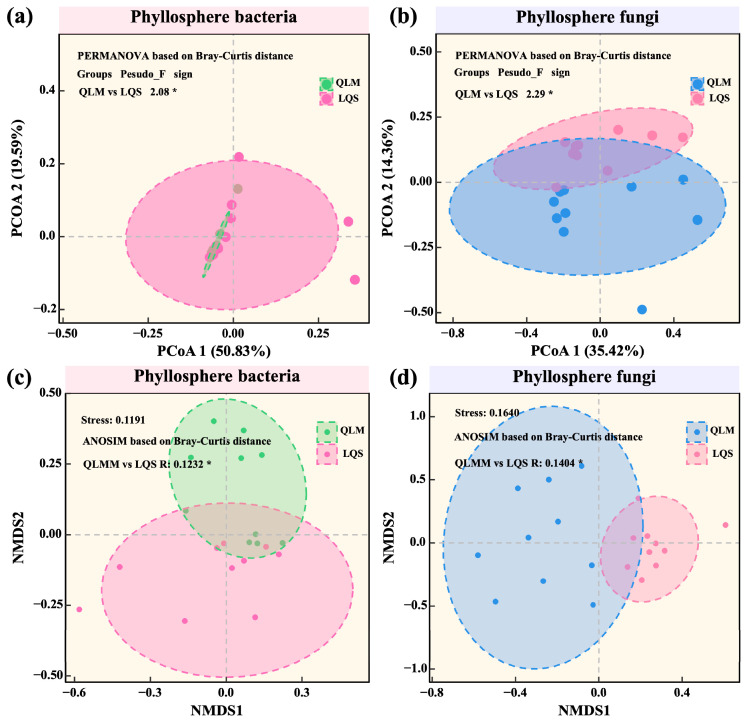
Comparative analysis of phyllosphere bacterial and fungal community structures between QLM and LQS. (**a**) Phyllosphere bacterial communities. (**b**) Phyllosphere fungi communities. (**c**) Phyllosphere bacterial communities. (**d**) Phyllosphere fungi communities. Note: Pink background in the title indicates phyllosphere bacteria; blue background indicates phyllosphere fungi. Asterisks indicate statistical significance (* *p* < 0.05).

Beta diversity was assessed using Principal Coordinates Analysis (PCoA) based on weighted UniFrac distances and Non-Metric Multidimensional Scaling (NMDS) based on Bray–Curtis dissimilarities. For bacterial communities, PCoA revealed clear spatial separation along the PCoA1 (50.83%) and PCoA2 (19.59%) axes, yielding significant differences between sites (PERMANOVA, pseudo-F = 2.08, *p* < 0.05; Figure 4a). This result was further supported by NMDS (ANOSIM, R = 0.1232, *p* < 0.05; Figure 4c). In comparison, geographic divergence was even stronger in fungal communities: significant separation was detected by PCoA (pseudo-F = 2.29, *p* < 0.05; Figure 4b) and NMDS (ANOSIM, R = 0.1404, *p* < 0.05; Figure 4d). Taken together, these analyses indicate that environmental gradients exert a greater influence than host identity on phyllosphere microbial β-diversity in alpine steppes.

To assess the influence of environmental factors on phyllosphere microbial diversity, Mantel tests were used to quantify the relationships between microbial α- and β-diversity and climate, vegetation, and soil variables.

**Figure 5:**
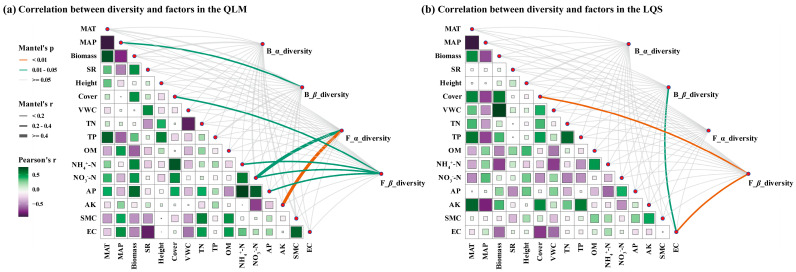
Mantel test analysis of bacterial and fungal α and β diversity in phyllosphere communities between QLM and LQS. (**a**) Phyllosphere microbiome diversity in the QLM. (**b**) Phyllosphere microbiome diversity in the LQS. Bacterial α diversity (B_α_diversity), Bacterial β diversity (B_β_diversity), Fungal α diversity (F_α_diversity), Fungal β diversity (F_β_diversity). Note: Detailed Mantel test statistics for the correlations between microbial diversity indices and environmental variables are provided in Supplementary Tables S1 and S2.

At QLM (Figure 5a; Supplementary Table S1), bacterial α-diversity was not significantly linked to any environmental variable (*p* > 0.05; Figure 5a; Supplementary Table S1), while β-diversity showed a significant association with MAP (r = 0.220, *p* < 0.05; Figure 5a; Supplementary Table S1). In contrast, fungal communities were more responsive to environmental variation: α-diversity correlated with soil NO_3_^−^-N (r = 0.448, *p* < 0.05; Figure 5a) and displayed a strong relationship with AK (r = 0.710, *p* < 0.01; Figure 5a; Supplementary Table S1). Fungal β-diversity was positively related to vegetation cover (r = 0.282, *p* < 0.05; Figure 5a; Supplementary Table S1), soil NH_4_^+^–N (r = 0.306, *p* < 0.05; Figure 5a; Supplementary Table S1), soil NO_3_^−^–N (r = 0.348, *p* < 0.05; Figure 5a; Supplementary Table S1), and AP (r = 0.339, *p* < 0.05; Figure 5a; Supplementary Table S1).

At LQS (Figure 5b; Supplementary Table S2), bacterial α-diversity again lacked significant correlations, but β-diversity was significantly correlated with EC (r = 0.393, *p* < 0.05; Figure 5b; Supplementary Table S2), indicating the importance of soil salinity in shaping bacterial spatial heterogeneity. Fungal α-diversity showed no meaningful associations, but β-diversity was strongly correlated with vegetation cover (r = 0.300, *p* < 0.05; Figure 5b; Supplementary Table S2) and EC (r = 0.390, *p* < 0.05; Figure 5b; Supplementary Table S2), suggesting that fungal community structure is jointly shaped by vegetation and soil salinity.

Collectively, these results indicate that bacterial communities at QLM are mainly governed by precipitation, while fungal communities respond predominantly to soil nutrients and vegetation structure. In LQS, soil salinity emerged as the primary determinant of β-diversity, with vegetation cover further influencing fungal community composition. Taken together, the contrasting drivers reveal distinct ecological strategies adopted by phyllosphere bacteria and fungi across alpine steppe habitats.

### 3.3. Host and Environmental Factors Shape Assembly of Phyllosphere Microbial Communities

**Figure 6:**
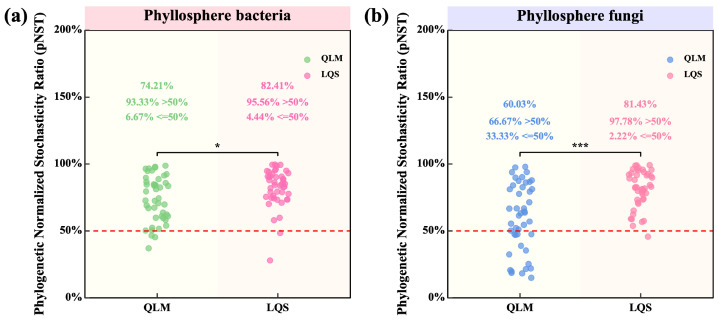
Comparative analysis of bacterial and fungal Phylogenetic Normalized Stochasticity Ratio (pNST) in phyllosphere communities between QLM and LQS. The values in figures (**a**,**b**) indicate means. Pairwise comparisons among treatments QLM (*n* = 45) and LQS (*n* = 45) were conducted using independent two-sample *t*-tests (df = 88), and final P-values were adjusted using the Bonferroni correction. Note: Pink background in the title indicates phyllosphere bacteria; blue background indicates phyllosphere fungi. Asterisks indicate statistical significance (* *p* < 0.05; *** *p* < 0.001).

To quantify the relative contributions of stochastic and deterministic processes in phyllosphere community assembly, we calculated the phylogenetic normalized stochasticity ratio (pNST) for bacterial and fungal communities at both sites. pNST values were significantly higher at LQS than at QLM, indicating a stronger stochastic signal at LQS (Figure 6).

For bacteria, 82.41% of LQS samples had pNST > 0.5 (Figure 6a), compared with 74.2% at QLM (*p* < 0.05). This contrast was even larger for fungi: 81.43% of LQS samples exceeded the threshold, whereas only 60.0% did so at QLM (*p* < 0.001; Figure 6b). Moreover, nearly all samples at LQS surpassed this threshold (95.6% for bacteria and 97.8% for fungi), underscoring the predominance of stochastic processes at this site.

Collectively, these findings suggest that the greater environmental heterogeneity and limited dispersal characteristic of LQS promote a more stochastic assembly of phyllosphere communities in the alpine steppe.

**Figure 7:**
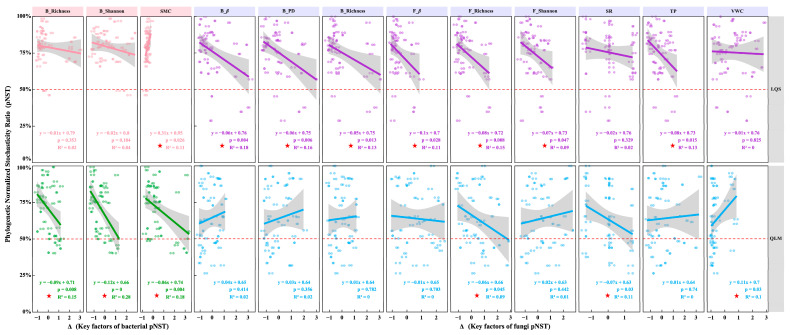
Driving factors of bacterial and fungal phylogenetic normalized stochasticity ratio (pNST) in phyllosphere communities between QLM and LQS. Note: Red pentagrams denote key indicators showing statistically significant differences. Pink background in the title indicates phyllosphere bacteria; blue background indicates phyllosphere fungi.

At the LQS site (Figure 7), phyllosphere bacterial pNST was positively correlated with soil moisture variation (ΔSMC; *p* = 0.026, R^2^ = 0.11), indicating that greater heterogeneity in water availability enhances the stochastic assembly of bacterial communities. In contrast, fungal pNST showed significant negative relationships with bacterial β-diversity (ΔB_β), bacterial phylogenetic diversity (ΔB_PD), bacterial richness (ΔB_Richness), fungal β-diversity (ΔF_β), fungal richness (ΔF_Richness), fungal Shannon diversity (ΔF_Shannon), and soil total phosphorus (ΔTP) (*p* < 0.05; Figure 7), suggesting that rising microbial diversity and nutrient supply strengthen deterministic control over fungal assembly.

At the QLM site, bacterial pNST was significantly negatively correlated with bacterial richness (ΔB_Richness), bacterial Shannon diversity (ΔB_Shannon), and soil moisture variation (ΔSMC) (*p* < 0.05; Figure 7), implying that both high microbial diversity and more stable hydrological conditions promote deterministic bacterial assembly. For fungal communities, pNST was negatively related to fungal richness (ΔF_Richness) and plant species richness (ΔSR), while positively correlated with variation in volumetric water content (ΔVWC) (*p* < 0.05; Figure 7), demonstrating opposing effects of biotic diversity and environmental heterogeneity on fungal assembly processes.

Together, these findings highlight taxon-specific assembly rules and underscore the influence of microbial diversity, vegetation structure, and soil properties on phyllosphere communities within alpine steppe ecosystems.

## 4. Discussion

### 4.1. Regional Divergence of Alpine Phyllosphere Microbiota

The composition and diversity of phyllosphere microbial communities are shaped by a combination of host identity and environmental context and often follow biogeographic trends similar to those documented for macroorganisms [42]. Among these factors, geographic position and its associated environmental gradients are regarded as the principal forces structuring microbial assemblages; consequently, a single plant species may support markedly different communities in separate regions [20,43].

In this study, we profiled the predominant bacterial and fungal taxa at the phylum and genus levels at two alpine-steppe sites to evaluate regional divergence. Although the vegetation at both sites is comparable and the overall taxonomic backbone was broadly conserved, distinct inter-site patterns emerged. At the phylum level, the phyllosphere bacterial community was dominated by *Cyanobacteriota*, which accounted for more than 75% of the relative abundance at both sites. The prominence of this phylum likely reflects its efficient photoautotrophy and strong tolerance of intense ultraviolet radiation and drought, two characteristic stresses of the alpine steppe, underscoring its ecological importance in this biome [44]. At the genus level, a sizeable proportion of sequences remained unclassified; nevertheless, classified genera such as *Scytonema* and *Exiguobacterium* exhibited high environmental adaptability and are known to engage in beneficial plant–microbe interactions [45,46], suggesting that they may contribute to microbial shielding or facilitate nutrient acquisition on leaf surfaces.

Fungal communities were dominated by *Ascomycota*, representing more than 98% of all sequences at the LQS site. At the genus level, *Epichloë*, *Cladosporium*, and *Fusarium* were comparatively enriched at QLM, while *Aspergillus* and *Alternaria* predominated the LQS site. These compositional differences probably reflect regional climatic drivers such as precipitation and temperature, which modulate phyllospheric microhabitats and host physiology and thereby favor taxa adapted to local conditions. Taken together, our results show that although phyllosphere microbial communities in alpine steppes are broadly similar at higher taxonomic ranks, they diverge markedly at the genus level, underscoring the combined roles of host filtering and environmental selection in structuring community composition [15,47].

### 4.2. Host–Environment Interplay Shapes Phyllosphere Diversity Patterns in Alpine Steppes

We systematically compared phyllosphere microbial α- and β-diversity between two alpine steppe sites (QLM and LQS), and identified the key environmental factors that shape these patterns. Although both sites harbored largely comparable vegetation, microbial communities showed pronounced spatial heterogeneity, indicating that abiotic factors may play a greater role than host filtering in structuring community composition. For α-diversity, species richness, Shannon index, and phylogenetic diversity did not differ significantly between sites for either bacterial or fungal communities. These suggest that under comparable vegetation composition and functional group structure, host plants may impose a consistent filtering effect on phyllosphere microbial α-diversity, while the influence of environmental variability remains relatively limited, especially at the level of species composition [48].

In contrast, β-diversity analyses revealed marked regional differentiation. PCoA and NMDS both indicated distinct clustering patterns between QLM and LQS, a result further confirmed by PERMANOVA and ANOSIM. Notably, fungal communities exhibited greater spatial differentiation than bacterial communities, likely due to their relatively larger propagules and reduced dispersal capacity, making them more sensitive to environmental heterogeneity [49].

The Mantel test clarified how diversity responds to environmental drivers. At QLM, bacterial α-diversity correlated solely with mean annual precipitation. In contrast, fungal α- and β-diversity were significantly correlated with multiple variables, including soil nitrate-N, available potassium, plant cover, available phosphorus, and ammonium-N. These results underscore the greater sensitivity of fungal communities to nutrient availability and vegetation structure [50], likely reflecting their resource-acquisition strategies and narrower habitat preferences. At LQS, soil electrical conductivity (EC) was the primary driver of both bacterial and fungal β-diversity, suggesting that salinity represents a key environmental determinant of microbial spatial heterogeneity [51]. In addition, fungal β diversity at LQS was significantly shaped by plant cover, emphasizing the importance of vegetation–soil interactions for fungal communities [50].

In summary, phyllosphere bacterial β-diversity is mainly influenced by precipitation and soil salinity, while fungal communities show greater sensitivity to soil nutrient availability and vegetation structure. This divergence reflects fundamental differences between the two microbial groups in their ecological niche adaptation and resource utilization strategies.

### 4.3. Host and Environmental Drivers of Phyllosphere Community Assembly

Community assembly describes how species interact through ecological processes, adapt to their environment, and establish stable, coexisting communities. These processes are generally classified as deterministic or stochastic, and together they shape the structure and dynamics of microbial communities [52]. In this study, the phylogenetic normalized stochasticity ratio (pNST) was used to quantify the relative influence of these forces on phyllosphere microbial community assembly at two alpine steppe sites (QLM and LQS), and environmental variables were included to identify the ecological drivers of assembly. The results revealed marked spatial heterogeneity, with stochastic processes predominating at the LQS site.

In general, pNST values for both bacterial and fungal communities were significantly higher at LQS than at QLM, and this contrast was most pronounced for fungi, with 81.43% of LQS exhibiting pNST values above 0.5, compared to 60.03% at QLM. This difference likely reflects greater environmental heterogeneity, dispersal limitation, and microclimatic variability at LQS, which weaken deterministic species selection and amplify stochastic influences, consistent with patterns reported in alpine ecosystems where bacterial assembly often mirrors dispersal limitation and spatial patchiness [53].

Further analysis at the LQS site showed that fluctuations in soil moisture content (ΔSMC) exerted a significant positive influence on bacterial pNST, corroborating observations that episodic water availability promotes stochastic assembly by modulating bacterial dispersal and colonization [54]. In contrast, fungal pNST was negatively associated with several community-level metrics, including bacterial and fungal beta diversity, phylogenetic diversity, species richness, Shannon diversity, and total soil phosphorus. These relationships indicate that increasing heterogeneity in community structure or nutrient status strengthens environmental filtering and reduces the relative influence of stochastic processes. Similar patterns have been reported for soil microbiomes, where greater phosphorus availability reinforces deterministic assembly in rhizosphere communities [55].

At QLM, bacterial pNST was significantly negatively correlated with bacterial community richness, diversity, and soil moisture, suggesting that deterministic forces, notably environmental selection, dominate when conditions are comparatively stable; this pattern echoes observations in wetland microbiomes [56]. Similarly, fungal pNST was negatively linked to plant species richness and fungal diversity, underscoring the role of a consistent host assemblage and habitat structure in steering fungal community assembly [55]. By contrast, the positive relationship between plant volumetric water content (ΔVWC) and fungal pNST indicates that fluctuations in moisture weaken environmental filtering and enhance stochasticity [55,56].

Taken together, these findings show clear divergence in phyllosphere assembly mechanisms between the two alpine steppe sites. Stochastic processes prevail at LQS, driven by site-specific patterns of diversity, soil moisture, and nutrient heterogeneity. Bacterial communities respond mainly to moisture variability, whereas fungal communities are more sensitive to shifts in diversity and vegetation structure.

## 5. Conclusions

This study compared two alpine steppe sites on the Qinghai–Tibet Plateau, the QLM and LQS, which possess similar vegetation yet experience contrasting long-term climates marked by differences in precipitation and temperature. We systematically investigated phyllosphere microbial composition, diversity patterns, and community assembly processes to assess the relative influence of environmental variables and host vegetation at the plant-community level. Under similar host conditions, α-diversity showed no significant variation between the sites. However, significant geographic differentiation emerged in species composition, β-diversity, and community assembly mechanisms of phyllosphere microbial communities. At the LQS site, community assembly was largely driven by stochastic processes and strongly associated with variability in moisture, soil salinity, and nutrient availability. In comparison, although stochastic processes also dominated at the QLM site, deterministic processes played a relatively greater role, suggesting that stronger host filtering and more stable environmental conditions imposed enhanced deterministic influences on community assembly. These findings further confirm that phyllosphere microbial communities are jointly shaped by environmental and host factors, and underscore their pronounced sensitivity to environmental disturbances under climate change. Overall, these results provide empirical evidence of plant–phyllosphere microbial interactions in alpine steppe ecosystems and offer a theoretical framework to predict and manage phyllosphere microbial diversity and functional stability under extreme environmental conditions.

## Figures and Tables

**Table 1:** Differences in environmental and plant-related factors between LQS and QLM.

Environmental and Plant	Factors	Qinghai Lake Margin Site (LQS)	Qilian Mountains Site (QLM)
Climatic Factors	MAT (mm)	0.61 ± 0.03 a	−3.91 ± 0.06 b
	MAP (°C)	457.28 ± 0.26 a	330.92 ± 1.53 b
Plant Traits	Biomass (g/m^2^)	61.44 ± 5.40 a	52.41 ± 2.37 a
	Species richness	5.11 ± 0.88 a	4.20 ± 0.79 b
	Height (cm)	6.42 ± 1.52 b	8.24 ± 1.73 a
	Cover (%)	86.41 ± 5.04 a	80.7 ± 6.62 a
	VWC (g/m^2^)	9.44 + 2.35 a	2.82 ± 1.95 b
	TN (mg/kg)	2175.97 ± 412.97 a	2097.32 ± 258.84 a
	TP (mg/kg)	295.27 ± 40.11 a	335.46 ± 59.17 a
	OM (%)	5.01 ± 0.67 a	4.16 ± 0.63 b
Soil	NH_4_^+^-N (mg/kg)	5.46 ± 1.96 b	8.18 ± 2.25 a
Physicochemical	NO_3_^−^-N (mg/kg)	22.39 ± 2.23 a	31.07 ± 4.87 a
Properties	AP (mg/kg)	6.71 ± 2.35 a	6.11 ± 2.14 a
	AK (mg/kg)	202.78 ± 84.88 a	197.73 ± 26.82 a
	SMC (%)	36.15 ± 3.75 a	33.38 ± 7.48 a
	EC (us/cm)	0.38 ± 0.11 a	0.99 ± 0.24 a

Kruskal–Wallis tests: MAT, MAP, Biomass, Species richness, VWC, TN, TP, NO_3_^−^-N, AK, SMC, EC. Two-sample *t*-tests: Height, Cover, OM, NH_4+_-N, AP. mean annual temperature, MAT; mean annual precipitation, MAP; above-ground biomass, Biomass; species richness, SR; volumetric water content, VWC; total nitrogen, TN; total phosphorus, TP; nitrate nitrogen, NO_3_^−^-N; available potassium, AK; soil moisture content, SMC; and electrical conductivity, EC; plant height, Height; canopy cover, Cover; soil organic matter, OM; ammonium nitrogen, NH_4+_-N; and available phosphorus, AP. Lowercase letters (a, b) indicate significant differences between LQS and QLM at the 0.05 level. Values with the same letter are not significantly different. Note: Blue represents Climatic Factors, Green represents Plant Traits, and Brown represents Soil Physicochemical Properties in the table.

## Data Availability

Data is deposited in the National Microbiology Data Center (NMDC) with accession numbers NMDC40084200 (https://nmdc.cn/resource/genomics/metagenome/detail/NMDC40084200, accessed on 15 April 2025).

## Supplementary Materials

The following supporting information can be downloaded at https://www.mdpi.com/article/10.3390/microorganisms13061432/s1. Table S1: Mantel test results for the correlations between microbial diversity and factors at the QLM site. Table S2: Mantel test results for the correlations between microbial diversity and factors at the LQS site.
