# Supplementary material for: Host-Determined Diversity and Environment-Shaped Community Assembly of Phyllosphere Microbiomes in Alpine Steppes Ecosystems
Source: Microorganisms. 2025 Jun 19;13(6):1432. doi: 10.3390/microorganisms13061432 (PMC12195958; doi:10.3390/microorganisms13061432)
Supplement: Supplementary file 1 [file microorganisms-13-01432-s001.zip › microorganisms-3666326-supplementary.pdf]

Supplementary materials for

Host-Determined diversity and environment-shaped community assembly of phyllosphere microbiomes in alpine steppes ecosystems

Kaifu Zheng †, Xin Jin †, Jingjing Li , Guangxin Lu *

College of Agriculture and Animal Husbandry, Qinghai University, Xining 810016, China;
zhengkf@qhu.edu.cn (K.Z.); 18894310895@163.com (X.J.);
lijingjing1879705@163.com (J.L.)
* Correspondence: lugx74@163.com; Tel.: +86-13897216290
† These authors contributed equally to this work.

Table S1. Mantel test results for the correlations between microbial diversity and factors at the QLM site.

Variant1	Variant2	Spearman'r	p_value	Significance
B_α_diversity	MAT	0.101227178	0.26	ns
B_α_diversity	MAP	0.136762112	0.169	ns
B_α_diversity	Biomass	0.207666134	0.148	ns
B_α_diversity	SR	-0.19422863	0.941	ns
B_α_diversity	Height	0.152279437	0.15	ns
B_α_diversity	Cover	0.114493489	0.203	ns
B_α_diversity	VWC	-0.146662633	0.613	ns
B_α_diversity	TN	-0.095914908	0.627	ns
B_α_diversity	TP	0.125769228	0.241	ns
B_α_diversity	OM	-0.080012974	0.666	ns
B_α_diversity	NH_4^+-N	0.161779937	0.157	ns
B_α_diversity	NO_3^--N	0.293373172	0.066	ns
B_α_diversity	AP	0.020092172	0.42	ns
B_α_diversity	AK	0.067765524	0.323	ns
B_α_diversity	SMC	0.302147315	0.094	ns
B_α_diversity	EC	-0.13444047	0.872	ns
B_β_diversity	MAT	0.251797011	0.061	ns
B_β_diversity	MAP	0.219798443	0.048	*
B_β_diversity	Biomass	0.034956622	0.284	ns
B_β_diversity	SR	0.053752082	0.227	ns
B_β_diversity	Height	0.061179222	0.349	ns
B_β_diversity	Cover	-0.098746836	0.718	ns
B_β_diversity	VWC	-0.050791308	0.449	ns
B_β_diversity	TN	0.025246705	0.339	ns

B_β_diversity	TP	0.253343845	0.064	ns
B_β_diversity	OM	-0.003540307	0.48	ns
B_β_diversity	NH_4^+-N	-0.140057761	0.793	ns
B_β_diversity	NO_3^--N	0.006629593	0.354	ns
B_β_diversity	AP	-0.000633939	0.447	ns
B_β_diversity	AK	-0.042696439	0.538	ns
B_β_diversity	SMC	0.288624325	0.067	ns
B_β_diversity	EC	0.144182507	0.212	ns
F_α_diversity	MAT	-0.050957458	0.575	ns
F_α_diversity	MAP	0.046151864	0.384	ns
F_α_diversity	Biomass	0.163417477	0.236	ns
F_α_diversity	SR	-0.006735541	0.455	ns
F_α_diversity	Height	0.107290886	0.225	ns
F_α_diversity	Cover	-0.045115687	0.56	ns
F_α_diversity	VWC	-0.149948124	0.66	ns
F_α_diversity	TN	-0.217579414	0.878	ns
F_α_diversity	TP	0.154059096	0.211	ns
F_α_diversity	OM	-0.074980502	0.652	ns
F_α_diversity	NH_4^+-N	0.051101052	0.351	ns
F_α_diversity	NO_3^--N	0.448151153	0.021	*
F_α_diversity	AP	0.031441457	0.406	ns
F_α_diversity	AK	0.710471293	0.002	**
F_α_diversity	SMC	-0.231592898	0.974	ns
F_α_diversity	EC	-0.162876123	0.925	ns
F_β_diversity	MAT	-0.21626921	0.968	ns
F_β_diversity	MAP	-0.160103639	0.947	ns
F_β_diversity	Biomass	0.118032647	0.191	ns
F_β_diversity	SR	-0.203713634	0.902	ns
F_β_diversity	Height	0.122018518	0.243	ns
F_β_diversity	Cover	0.281678858	0.029	*
F_β_diversity	VWC	-0.037525515	0.399	ns
F_β_diversity	TN	-0.117680242	0.762	ns
F_β_diversity	TP	0.147482157	0.154	ns
F_β_diversity	OM	0.020527123	0.389	ns
F_β_diversity	NH_4^+-N	0.306227003	0.04	*
F_β_diversity	NO_3^--N	0.348388701	0.012	*
F_β_diversity	AP	0.338552124	0.025	*
F_β_diversity	AK	0.039424663	0.362	ns
F_β_diversity	SMC	-0.159875942	0.923	ns
F_β_diversity	EC	-0.097835259	0.651	ns

Asterisks indicate the level of significance ($^{NS}p > 0.05$;$^{*}p < 0.05$;$^{**}p < 0.01$).

Table S2. Mantel test results for the correlations between microbial diversity and factors at the LQS site.

Variant1	Variant2	Spearman'r	p_value	Significance
B_α_diversity	MAT	-0.067233178	0.532	ns
B_α_diversity	MAP	-0.041447366	0.467	ns
B_α_diversity	Biomass	0.089491276	0.156	ns
B_α_diversity	SR	0.142784544	0.152	ns
B_α_diversity	Height	0.256311974	0.22	ns
B_α_diversity	Cover	-0.032050868	0.559	ns
B_α_diversity	VWC	0.035182343	0.32	ns
B_α_diversity	TN	-0.147943555	0.883	ns
B_α_diversity	TP	-0.033036863	0.493	ns
B_α_diversity	OM	-0.186359278	0.723	ns
B_α_diversity	NH_4^+-N	0.155747086	0.158	ns
B_α_diversity	NO_3^--N	-0.125022134	0.637	ns
B_α_diversity	AP	-0.066543933	0.563	ns
B_α_diversity	AK	-0.30025267	0.99	ns
B_α_diversity	SMC	-0.229766373	0.929	ns
B_α_diversity	EC	0.052874464	0.3	ns
B_β_diversity	MAT	-0.133032371	0.751	ns
B_β_diversity	MAP	-0.123619237	0.74	ns
B_β_diversity	Biomass	-0.371407686	0.999	ns
B_β_diversity	SR	-0.026661697	0.562	ns
B_β_diversity	Height	0.118199702	0.252	ns
B_β_diversity	Cover	0.037223723	0.382	ns
B_β_diversity	VWC	-0.457664563	1	ns
B_β_diversity	TN	-0.099643415	0.718	ns
B_β_diversity	TP	-0.280565377	0.967	ns
B_β_diversity	OM	0.100594115	0.261	ns
B_β_diversity	NH_4^+-N	-0.196553642	0.872	ns
B_β_diversity	NO_3^--N	-0.12033113	0.746	ns
B_β_diversity	AP	0.008323797	0.453	ns
B_β_diversity	AK	-0.157624734	0.791	ns
B_β_diversity	SMC	0.055943605	0.343	ns
B_β_diversity	EC	0.392905801	0.025	*
F_α_diversity	MAT	-0.195915426	0.795	ns
F_α_diversity	MAP	-0.183833723	0.799	ns
F_α_diversity	Biomass	-6.68E-05	0.423	ns
F_α_diversity	SR	-0.055894671	0.634	ns
F_α_diversity	Height	0.159546571	0.246	ns
F_α_diversity	Cover	0.025824887	0.361	ns
F_α_diversity	VWC	0.074814667	0.273	ns
F_α_diversity	TN	0.150358062	0.134	ns
F_α_diversity	TP	0.150490624	0.19	ns

F_α_diversity	OM	-0.113534418	0.667	ns
F_α_diversity	NH_4^+-N	0.011185051	0.399	ns
F_α_diversity	NO_3^--N	-0.018325113	0.465	ns
F_α_diversity	AP	-0.082841154	0.645	ns
F_α_diversity	AK	-0.298817067	0.993	ns
F_α_diversity	SMC	-0.037082654	0.548	ns
F_α_diversity	EC	0.144818343	0.211	ns
F_β_diversity	MAT	-0.146089516	0.756	ns
F_β_diversity	MAP	-0.143529127	0.768	ns
F_β_diversity	Biomass	0.105603023	0.293	ns
F_β_diversity	SR	0.162393406	0.256	ns
F_β_diversity	Height	-0.125680365	0.743	ns
F_β_diversity	Cover	0.299657937	0.006	**
F_β_diversity	VWC	0.016582171	0.396	ns
F_β_diversity	TN	-0.199737527	0.911	ns
F_β_diversity	TP	-0.150713787	0.818	ns
F_β_diversity	OM	-0.041842861	0.493	ns
F_β_diversity	NH_4^+-N	-0.027159367	0.491	ns
F_β_diversity	NO_3^--N	-0.117923558	0.716	ns
F_β_diversity	AP	-0.014315897	0.487	ns
F_β_diversity	AK	-0.137448142	0.779	ns
F_β_diversity	SMC	-0.092222155	0.679	ns
F_β_diversity	EC	0.390313373	0.006	**

Asterisks indicate the level of significance ($^{NS}p > 0.05$;*$p < 0.05$;**$p < 0.01$).
